# Two novel compound heterozygous mutations in *OPA3* in two siblings with OPA3-related 3-methylglutaconic aciduria

**DOI:** 10.1016/j.ymgmr.2014.02.003

**Published:** 2014-03-13

**Authors:** Christina Lam, Linda K. Gallo, Richard Dineen, Carla Ciccone, Heidi Dorward, George E. Hoganson, Lynne Wolfe, William A. Gahl, Marjan Huizing

**Affiliations:** a Medical Genetics Branch, National Human Genome Research Institute, National Institutes of Health, Bethesda, MD 20892, USA; b Department of Pediatrics, Edward Hospital, Naperville, IL, USA; c Department of Pediatrics, University of Illinois, Chicago, IL, USA

**Keywords:** 3-MGA-uria, 3-methylglutaconic aciduria, 3-MGA, 3-methylglutaconic acid, 3-MGR, 3-methylglutaric acid, MRI, magnetic resonance imaging, Costeff optic atrophy syndrome, Extrapyramidal dysfunction, 3-Methyl glutaconic aciduria, Mitochondrial pathology, OPA3, Optic atrophy plus syndrome

## Abstract

OPA3-related 3-methylglutaconic aciduria, or Costeff Optic Atrophy syndrome, is a neuro-ophthalmologic syndrome of early-onset bilateral optic atrophy and later-onset spasticity, and extrapyramidal dysfunction. Urinary excretion of 3-methylglutaconic acid and of 3-methylglutaric acid is markedly increased. OPA3-related 3-methylglutaconic aciduria is due to mutations in the *OPA3* gene located at 19q13.2–13.3. Here we describe two siblings with novel compound heterozygous variants in *OPA3*: c.1A>G (p.1M>V) in the translation initiation codon in exon 1 and a second variant, c.142+5G>C in intron 1. On cDNA sequencing the c.1A>G appeared homozygous, indicating that the allele without the c.1A>G variant is degraded. This is likely due to an intronic variant; possibly the IVS1+5 splice site variant. The older female sibling initially presented with motor developmental delay and vertical nystagmus during her first year of life and was diagnosed subsequently with optic atrophy. Her brother presented with mildly increased hip muscle tone followed by vertical nystagmus within the first 6 months of life, and was found to have elevated urinary excretion of 3-methylglutaconic acid and 3-methylglutaric acid, and optic atrophy by 1.5 years of age. Currently, ages 16 and 7, both children exhibit ataxic gaits and dysarthric speech. Immunofluorescence studies on patient's cells showed fragmented mitochondrial morphology. Thus, though the exact function of OPA3 remains unknown, our experimental results and clinical summary provide evidence for the pathogenicity of the identified OPA3 variants and provide further evidence for a mitochondrial pathology in this disease.

## 1 Introduction

The branched-chain organic acids 3-methylglutaconic acid (3-MGA) and 3-methylglutaric acid (3-MGR) are excreted only in trace amounts in the urine of healthy individuals (less than ~ 6 mmol/mol creatinine) [1]. Moderately increased urinary excretion of 3-MGA and 3-MGR (20–40 mmol/mol creatinine) is a relatively common finding in metabolic disorders, especially in disorders with mitochondrial dysfunction, and is often accompanied by increased excretion of other (disease specific) metabolites. 3-Methylglutaconic acidurias (3-MGA-urias) represent a group of disorders with significantly and consistently increased urinary 3-MGA and 3-MGR (40–1000 mmol/mol creatinine), where these increased metabolites are a hallmark of the phenotype and the key to diagnosis of the disorder [2], [3]. A group of distinct clinical syndromes of 3-MGA-urias have been described [3], [4].

**Fig. 1:**
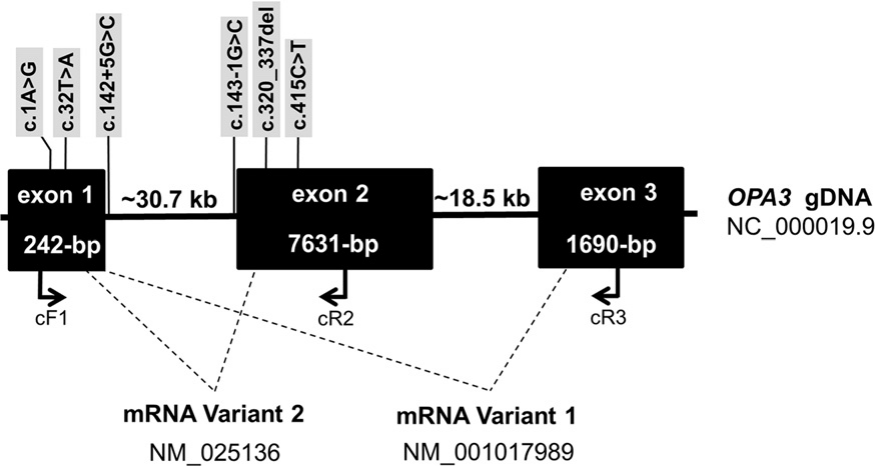
Structure of the *OPA3* gene and OPA3-related 3-MGA-uria sequence variants. Schematic of the *OPA3* locus on chromosome 19q13.32 (not to scale). Introns (black lines), exons (black boxes), the two mRNA splice variants and locations and directions of primers used to amplify variant-specific cDNA fragments are indicated. *OPA3* sequence variants associated with OPA3-related 3-MGA-uria are indicated in gray highlight; note that all reported variants occur in exons 1 or 2 (mRNA Variant 2).

OPA3-related 3-MGA-uria, or Costeff optic-atrophy syndrome (former type III 3-MGA-uria; MIM 258501), is characterized by early bilateral optic atrophy with decreased visual acuity and later development of spastic paraparesis, mild ataxia, and occasionally cognitive deficit in the second decade. Following the development of these symptoms, the course of the disease is relatively stable [5], [6]. OPA3-related 3-MGA-uria is inherited in an autosomal recessive pattern and due to mutations in the *OPA3* gene [7]. The exact underlying pathology of the disease and of OPA3 function remain elusive but is likely associated with mitochondrial dysfunction [8]. We previously described that the *OPA3* gene contains three exons encoding two transcripts formed by alternative splicing (Fig. 1) [8], which were recently annotated into GenBank (http://www.ncbi.nlm.nih.gov/genbank/). The longest *OPA3* mRNA transcript, variant 1, consists of exons 1 and 3 (GenBank NM_001017989), and the shorter mRNA variant 2 consists of exons 1 and 2 (NM_025136). The variant 1 (exons 1+3) *OPA3* mRNA transcript has lower expression than variant 2 (exons 1+2), and mRNA variant 1 may not yield a significant translation product in human cells, since its protein product is not identified in proteomic databases and no human disease has been associated with mutations in the variant 1-specific exon 3 [8]. In addition, variant 2 (exons 1+2) is expressed and conserved from fungi to primates, while variant 1 is uniquely found in mammals. Both OPA3 protein products (products of mRNA variant 1, confusingly called OPA3A in GenBank and OPA3B in Huizing et al.; and of mRNA variant 2, called OPA3B in GenBank and OPA3A in Huizing et al. contain an N-terminal mitochondrial leader sequence and targeting signal and a putative C-terminal peroxisomal targeting signal [8].

The cellular role of OPA3 and its role in OPA3-related 3-MGA-uria pathology remains unknown; however, the presence of the N-terminal mitochondrial targeting sequences and the presence of OPA3 in mitochondrial protein databases (MITOP: http://78.47.11.150:8080/mitop2/, Mitoproteome: http://www.mitoproteome.org/, Mitominer: http://mitominer.mrc-mbu.cam.ac.uk/) strongly suggest mitochondrial involvement. Proteomic databases did not identify OPA3 as a peroxisomal protein (PeroxisomeDB, http://www.peroxisomeDB.org) [9]. In addition, cellular studies showed that OPA3 predominantly localized to mitochondria, that OPA3 is anchored to mitochondrial membranes and that overexpression or downregulation of *OPA3* led to altered mitochondrial morphology [10]. Moreover, mitochondrial involvement can explain the combination of elevated 3-MGA and 3-MGR [2] and optic maldevelopment and/or atrophy [11], [12] in patients. These findings thus placed the cellular metabolic defect of OPA3-related 3-MGA-uria in the mitochondrion.

So far, only a few *OPA3* mutations associated with OPA3-related 3-MGA-uria have been described (Table 1). Anikster et al. initially described a splice site mutation c.143-1G>C [IVS1-1G>C], in an Iraqi-Jewish cohort [7]. Subsequently only three other mutations were reported; a homozygous deletion c.320_337del [p.Q108_E113del] in exon 2 in a Kurdish-Turkish patient [13], a homozygous nonsense mutation in exon 2 at c.415C>T [p.Q139X] in an individual of Indian origin [14], and a homozygous missense mutation in exon 1 at c.32T>A [p.L11Q] in a Pakistani subject [15].

Of note, a few dominant inherited *OPA3* variants, p.G93S, p.Q105E, and p.V3_G4insAP result in a rare dominant disorder (ADOAC; MIM 165300) involving optic atrophy, cataracts and extrapyramidal signs [16], [17], [18]. The ADOAC phenotype may reflect a dominant negative effect, since heterozygous carriers of the Iraqi-Jewish loss of function founder mutation (c.143-1G>C) do not show a clinical phenotype. Similarly, a recently reported murine model harboring p.L122P in the heterozygous state appears normal [19].

Here we describe identification of two siblings with OPA3-related 3-MGA-uria who showed unique compound heterozygous variants of *OPA3*; a missense variant c.1A>G [p.1M>V] in the translation initiation codon and an intronic variant c.142+5G>C [IVS1+5G>C]. We utilized the patients' fibroblasts to assess the effect of these variants on *OPA3* mRNA and on mitochondrial morphology by immunocytochemistry. These studies reiterate the clinical phenotype and that the basic defect of OPA3-related 3-MGA-uria likely lies in the mitochondrion.

## 2 Methods

### 2.1 Patients and cells

Patient samples were enrolled under the NIH protocol “Diagnosis and Treatment of Patients with Inborn Errors of Metabolism” (http://clinicaltrials.gov/, trial NCT00369421), approved by the National Human Genome Research Institute's Institutional Review Board. Each patient or a parent gave written informed consent, in accordance with the Declaration of Helsinki. Genomic DNA was extracted from peripheral leukocytes using standard protocols from both patients. Skin fibroblasts were grown from a punch biopsy from Patient 2 according to standard protocols in Dulbecco's modified Eagle medium supplemented with 10% fetal bovine serum containing 100 U/ml penicillin and 0.1 mg/ml streptomycin. DNA, cDNA and cell imaging results in this study are displayed only for Patient 2 (Pt. 2). Patient 1 (Pt. 1) was found to have the same DNA variants as her brother, but we had no cDNA or cells available from her.

### 2.2 Molecular analysis

Primers were designed to amplify the three *OPA3* exons and their intronic boundaries from genomic DNA as described [8]. Standard PCR amplification procedures were employed. All amplified products were directly sequenced using the BigDye 3 Terminator chemistry (Applied Biosystems, Foster City, CA) and separated on an ABI 3130xl genetic analyzer (Applied Biosystems). RNA was isolated from cultured fibroblasts using the Trizol reagent (Invitrogen Life Technologies, Carlsbad, CA), and reversely transcribed into cDNA using the SuperScript First-Strand Synthesis System for Reverse Transcription-Polymerase Chain Reaction (Invitrogen Life Technologies, Carlsbad, CA). Primers were designed to distinguish between the two OPA3 mRNA splice variants, variant 1 (NM_001017989) and variant 2 (NM_025136) (Fig. 1); a common forward primer in exon 1 (cF1: 5′-GCAAGGTTGCGCGTGCCCTGTGAG-3′) was combined with a reverse primer specific for exon 2 (cR2: 5′-GGCCACGTTAGGTACATAGGCCATG-3′) to amplify *OPA3* variant 2 (625-bp fragment), or with a reverse primer specific for exon 3 (cR3: 5′-GTTCCACCTGCAGGAGGCGGA-3′) to amplify *OPA3* variant 1 (795-bp fragment). PCR amplifications were performed under standard conditions.

### 2.3 Immunocytofluorescence

Fibroblasts were grown overnight on slides (Laboratory-Tek, Nalge Nunc Int.), followed by treatment with MitoTracker Green (Molecular Probes) and fixation in 3% paraformaldehyde/phosphate-buffered saline (PBS). The slides were then blocked/permeabilized in PBS containing 0.1% saponin, 100 μM glycine, 0.1% BSA and 2% donkey serum followed by incubation with rabbit PMP-70 peroxisomal antibodies (Zymed). The cells were then washed and incubated with Cy5-labeled secondary antibodies conjugated (Molecular Probes), washed again, and mounted in VectaShield (Vector Laboratories). The cells were imaged with a Zeiss LSM 510 META confocal laser-scanning microscope (Carl Zeiss, Microimaging Inc.) using a 488 nm Argon and a 543 nm HeNe laser. All the images were acquired using a Plan NeoFluar 40X/1.3 oil DIC or a Plan Apochromat 63X/1.4 oil DIC objective.

## 3 Results

### 3.1 Clinical report

Patient (Pt.) 1, a 16 year old female, and her 7 year old brother (Pt. 2), were born to parents of Indian ancestry. Both parents are from southern India. The father originates from the town of Coimbatore and the mother is from Coonoor. Pt. 1 was born full-term by normal spontaneous vaginal delivery to a 19 year old mother in India. Birth weight was 2.8 kg (normal range: 2.5–4.2 kg). Motor delay was apparent in infancy; the girl did not walk until after 2 years of age. Vertical nystagmus was present before one year of age and optic atrophy was diagnosed based on the findings of optic disc pallor and nystagmus. Speech development was age appropriate. By 2 years of age the family moved to the USA and she was evaluated for a chief complaint of ataxia. At age 2 1/2 a brain magnetic resonance image (MRI) showed a slight prominence of signals in the white matter of the occipital and parietal lobes thought to be due to enhanced terminal myelination rather than leukomalacia. The brain MRI report was otherwise negative except for mild sinusitis. The images were not available for review. Mitochondrial studies performed on a skeletal muscle biopsy at age 3 showed no deficiency in the activity of the electron transport chain complexes. Surgical pathology reported type 1 fiber predominance with increased mitochondrial numbers and reactivity. Electron microscopy showed some enlarged mitochondria and others with an unusual shape. Acylcarnitine profile and carnitine levels were normal. Urine organic acid analysis conducted twice showed a small increase of 3-MGA, and OPA3-related 3-MGA-uria was tentatively diagnosed. Repeat brain MRI studies at ages 8 and 14 reported stable non-enhancing white matter signal hyperintensity in the peritrigonal white matter bilaterally. These MRI studies were optimized for brain, and not optic nerve structure; however, on a recent neuroradiology rereview the optic nerves were described as gracile. Other diagnostic studies included a normal complete metabolic panel and hemogram at age 12. Reduced and total coenzyme Q, carnitine profile, quantitative acylcarnitines, lactic acid, pyruvic acid, and plasma amino acids were normal at age 8. A repeat urine organic acid screen at age 8 revealed mildly elevated 3-MGA. At age 15 the urine organic acid analysis detected an elevated 3-MGA at 17 mmol/mol creatinine (normal range: less than 6 mmol/mol creatinine) and a normal 3-MGR of 0 mmol/mol creatinine.

Pt. 1 was enrolled in occupational and physical therapy from early childhood. By 4 years of age she was attending Montessori school and did well. Carnitine supplementation as part of a Mito Cocktail was attempted for one month but discontinued due to abdominal discomfort and no signs of improvement. At age 11 Pt. 1 developed tongue fasciculations and hirsutism. Menarche occurred at 13 years of age and menses were regular. Now 16 years old, this girl has a stable neurological impairment with ataxia, optic atrophy, nystagmus, legal blindness, and dysarthria. Leg braces and most recently a functional electrical stimulation unit have been used for foot drop and gait improvement. She has had several toe fractures due to falls. She attends a community school where she is a good student but needs an aide for visual assistance. She is in advanced placement geography. Occupational and physical therapy, vision services, aquatherapy, and mobility training are ongoing. In addition to the routine childhood illnesses, she has had chronic sinusitis.

On physical examination, patient 1 is a most pleasant young woman with height and weight at the 5%–10% and body mass index of 17 (normal range: 15–25). Vital signs are normal. There are no dysmorphic features. Dysconjugate eye movements are present with nystagmus. Speech is dysarthric with mild tongue fasciculations. Cardiorespiratory exam results are normal. There is no hepatosplenomegaly. She is ataxic with a wide-based, staggering, and unsteady gait. There are moderate to severe deficits in balance and coordination. The heel cords/hamstrings are tight; the hips are tight to a lesser degree. The spine shows minimal curvature. Overall strength is good. There is lower extremity hyperreflexia.

Pt. 2 was born full-term by normal spontaneous vaginal delivery and weighed 3.2 kg (normal range: 2.5–4.2 kg). Apgar scores were 8 and 9 with meconium-stained amniotic fluid. The nursery course was unremarkable. Both the hearing exam and the State of Illinois newborn screening results were normal. Due to his older sister's known diagnosis he was followed closely for the onset of symptoms. The first sign occurred when at 2 months of age his hip muscles were tight on abduction. An ophthalmology exam was normal. However, by 4–6 months of age vertical nystagmus developed. An MRI optimized for brain imaging was reported as normal but in that study the optic nerve was not evaluated directly. In re-examination, a neuroradiologist now describes the optic nerve proximal interconal segment as thin. The child was enrolled in Early Intervention Services. At 8 months of age a urine organic acid screen showed mildly elevated excretions of 3-MGA. By 1 1/2 years optic atrophy was present. The diagnosis was based on the presence of nystagmus and optic disc pallor. Like his sister he is legally blind. Weight gain decelerated after 6 months necessitating high calorie and protein supplementation. By 12 months his weight was less than the 5% but length and head circumference were preserved (both 50%). Like his sister, Pt. 2 displayed motor delay and did not walk until after 2 years of age at which time he was ataxic. He also had to use orthotics, splints, and braces for gait disturbance but he is ambulatory. In addition, speech delay was significant. At age 3, his comprehension was normal but expressive language was unclear and at a 15 month level. His second hearing exam results were normal. At the same age his weight began to increase to above 5%. Unlike his sister he has had recurrent skin infections. Quantitative immunoglobulins were normal or elevated. In addition, dental caries have been a concern and necessitated surgical extractions. Though his gait is markedly ataxic he has not sustained any fractures as a result of his many falls. Additional lab studies include a normal complete metabolic panel and hemogram. A repeat brain MRI at 6 years of age showed a few tiny nonspecific foci of increased T2/flair signal in bilateral parietal white matter questionable for mild leukoencephalopathy. Post inflammatory changes were noted in the paranasal sinuses, similar to his sister. The optic nerves were found to be atrophic on re-examination of the images by a neuroradiologist. At 7 years of age his 3-MGA was elevated at 24 mmol/mol creatinine (normal range: less than ~ 6 mmol/mol creatinine) and as his sister the 3-MGR was normal at 3 mmol/mol creatinine (normal range: less than ~ 3 mmol/mol creatinine).

This boy attends a community school where he receives special needs services for vision and speech. Parents report no intellectual deficit. At age 7, he is thin with weight at the 10% and height at the 25% with a body mass index of 14 (average range: 13–17). He is very active and often inattentive. Nystagmus persists and speech is dysarthric. Cardiorespiratory exam results are normal and no hepatosplenomegaly is present. Genitourinary exam reveals a prepubertal uncircumcised male. Congenital dermal melanocytosis lesions are present at the lumbosacral area and left ankle. The spine is straight. The hips are stable but straight leg raising is limited. Brisk reflexes and increased muscle tone are present in the lower extremities. His gait is ataxic. When ambulating he staggers, is unsteady, and has impaired postural control.

### 3.2 Molecular analysis

**Fig. 2:**
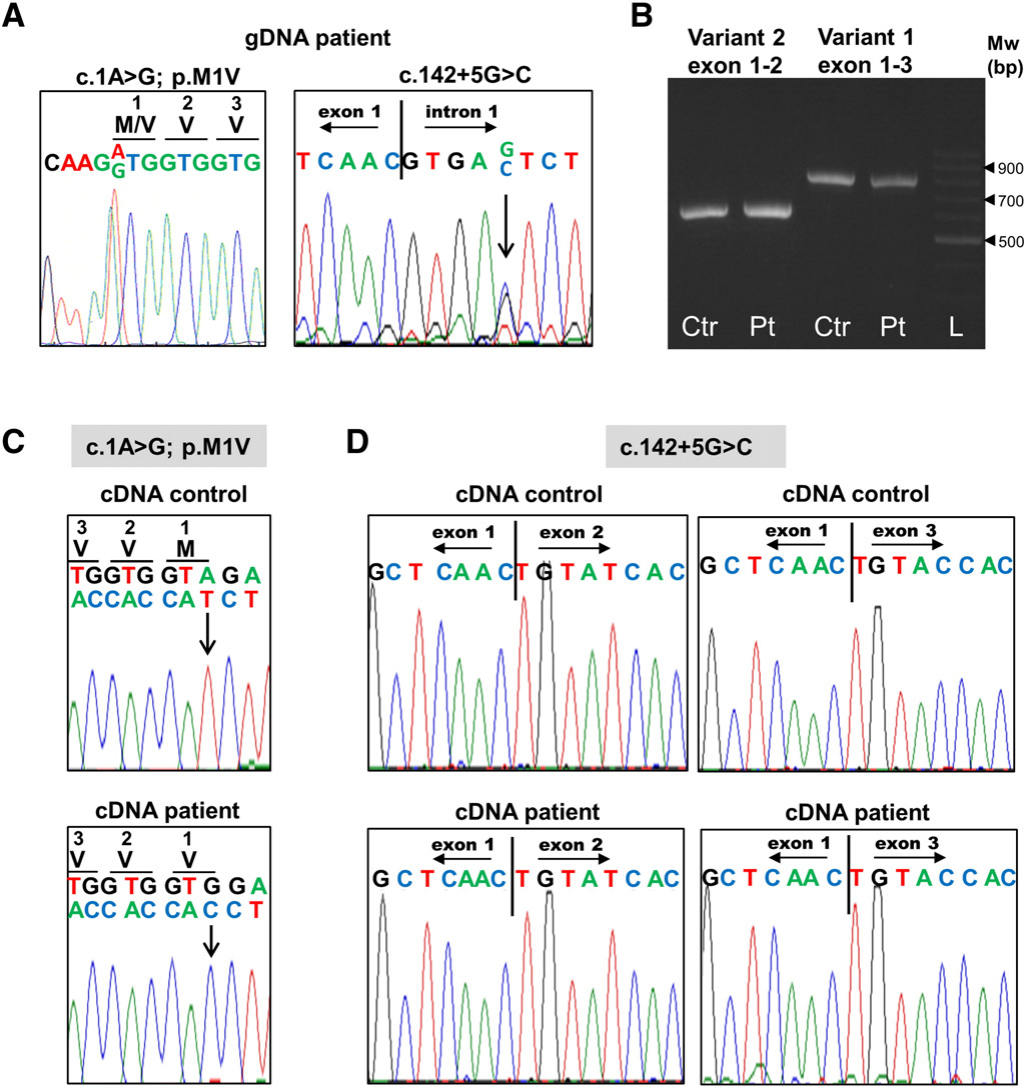
Molecular analysis. A. Genomic DNA sequence of Pt. 2 showing the heterozygous variants c.1A>G (exon 1) and c.142+5G>C (intron 1). B. PCR bands representing amplification of *OPA3* Variant 2 (primers cF1-cR2; 625-bp) and Variant 1 (primers cF1-cR3; 795-bp) did not markedly vary in size or concentration between control (Ctr) and Pt. 2 (Pt) fibroblast cDNA. C. Sequencing results (reverse sequence shown) over the c.1A>G variant from cDNA bands displayed in (B). Note that the patient's cDNA carries a homozygous change of c.1A>G, while the patient's gDNA carries this variant heterozygous (A).The patient's allele containing the adenosine at position c.1 may have undergone RNA decay. D. Sequencing results over the exon 1–2 (Variant 2) and exon 1–3 (Variant 1) splice region from cDNA bands displayed in (B). Both exon–exon boundaries have normal sequence in the patient's cDNA.

Our exon-specific genomic DNA analysis identified two novel *OPA3* variants in both siblings; a heterozygous missense variant in the translation initiation codon c.1A>G [p.1M>V] in exon 1, and a variant in intron 1 c.142+5G>C [IVS1+5G>C] (Fig. 2A). Splice variant-specific PCR amplification of *OPA3* from fibroblast cDNA (Pt. 2) yielded the same size and concentration of amplification products in the patient compared to the control (Fig. 2B); there were no additional (mis-spliced) bands present in the patient's sample. These results indicated that *OPA3* cDNA is produced in the patient's fibroblasts. Sequencing of the variant-specific cDNA bands from Fig. 2B, revealed that the c.1A>G variant appeared homozygous in the patient's cDNA, but was absent from control cDNA (Fig. 2C). This indicates that the mRNA allele containing the (wild type) adenosine nucleotide at position c.1 appeared decayed in the patient. Sequencing of the other cDNA bands from Fig. 2B did not reveal any other sequence variants in the patient compared to the control, including the sequences over the exon 1–2 and exon 1–3 splice regions (Fig. 2D), indicating that no (small) insertions or deletions took place in this region in the patient's cDNA. However, based on the sequencing results of Fig. 2C, it is likely that the cDNA PCR reaction (Fig. 2B) only amplified the mRNA allele containing the c.1A>G variant, which is expected to be normal in the exon 1–intron 1 splice region.

### 3.3 Cellular analysis

**Fig. 3:**
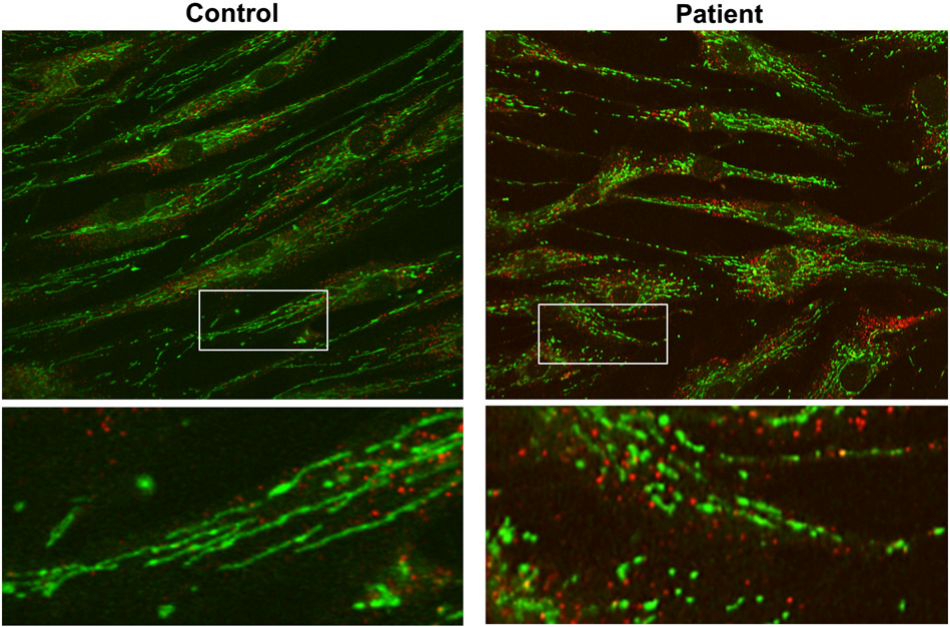
Cellular studies of mitochondria and peroxisomes in OPA3-related 3-MGA-uria. Mitochondria (green) and peroxisomes (red) in control and OPA3-related 3-MGA-uria (Pt. 2) fibroblasts were labeled with organelle-specific markers and imaged by confocal microscopy. Images are one-dimensional images of a Z-stack (Magnification: 40 ×). Bottom images are enlargements of boxed regions in upper images. While intracellular localization and distribution of peroxisomes and mitochondria in patient's fibroblasts appeared similar to the distribution in control fibroblasts, the morphology of the mitochondrial network was markedly fragmented in patient cells compared to control.

Since the OPA3 protein contains an N-terminal mitochondrial leader sequence and targeting signal and a putative C-terminal peroxisomal targeting signal [8], we studied whether OPA3 deficiency influenced the cellular distribution and abundance of these organelles in our patient's cells. Fluorescent labeling of mitochondria and peroxisomes revealed no apparent differences in the distribution of or abundance in fibroblasts from our OPA3-related 3-MGA-uria patient compared with control fibroblasts (Fig. 3). Both peroxisomes and mitochondria were uniformly distributed throughout the cells. However, while control fibroblasts showed an extensive tubular mitochondrial network, our patient's fibroblasts showed a markedly fragmented mitochondrial network, suggesting a disruption of mitochondrial morphology and dynamics similar to that seen in previous studies of cells with *OPA3* alterations [8], [10], [18].

## 4 Discussion

We identified two siblings with features of OPA3-related 3-MGA-uria. As a toddler, the presence of delayed motor development, nystagmus, and ataxia brought Pt. 1 to specialized medical attention that led to her diagnosis. Now in her second decade, she exhibits no evidence of intellectual decline and her neurological symptoms are stable. Except for their visual and motor difficulties, the children are of overall average health with no hospitalizations; they lead a mostly normal life. A diagnosis of 3 OPA3-related 3-MGA-uria was suspected based on the results of the urine organic acid screen and confirmed by DNA studies. The rarity of this illness and the unique OPA3 variants which these children have will make the clinical correlation and close observation of the natural history of this disease very important.

*OPA3* sequence analysis of the genomic DNA of both siblings identified two novel heterozygous variants associated with exon 1 of *OPA3*: c.1A>G [p.1M>V] and c.142+5G>C. The first variant yields a missense variant [p.1M>V] that likely precludes translation starting at the initial AUG sequence. Pathogenicity analysis of p.1M>V with in silico prediction software projected, as expected, this variant to be damaging (SIFT Genome), and probably damaging (PolyPhen-2). With truncation of the initiation codon, it is possible that a nearby downstream AUG sequence (in frame, c.22–24) is used as an alternative translation initiation codon, as has been described for other diseases with truncations in the translation start site [20], [21]. The translation software predicts the original initiation codon (c.1-3AUG) as 92% likely to be an initiation codon, and c.22–24AUG as only 42% likely. However, with the truncation of c.1A>G, codon c.22–24 is 77% likely to become the initiation codon (ATGpr; http://atgpr.dbcls.jp/). If c.22–24 becomes the alterative initiation codon, the alternative truncated N-terminal amino acid sequence (lacking the first 8 N-terminal amino acids compared to the wild type protein) is not predicted to be a mitochondrial leader sequence (MitoProt, http://ihg.gsf.de/ihg/mitoprot.html) and will therefore likely be non-functional. Unfortunately, no adequate working anti-human OPA3 antibodies were available to us to test protein size and expression by Western blotting and immunofluorescence in our patients' cells. Interestingly, on cDNA sequencing the c.1A>G variant appeared homozygous (Fig. 2C), indicating that the allele with the wild type sequence at c.1 is likely degraded. This could have occurred though nonsense-mediated RNA decay due to a severe truncating sequence variant on that allele.

The second variant in our patients, c.142+5G>C [IVS1+5G>C], is unlikely to result in alternative splicing but may reduce splicing at the exon 1–intron 1 junction, when evaluated by online splice site prediction software (http://www.fruitfly.org/seq_tools/splice.html). The wild type *OPA3* exon 1–intron 1 nucleotide sequence appears to have a 99% chance of being a splice donor site, while the variant splice site sequence containing c.142+5G>C in intron 1 has only a 31% likelihood of being a splice donor site. At a lower threshold, a new donor splice site 53-bp downstream of the exon/intron boundary is predicted to have only 15% likelihood of becoming a new splice donor site. Our PCR analysis of cDNA of both *OPA3* mRNA variants did not show any alternatively spliced bands (Fig. 2B), and the nucleotide sequence over the exon 1–2 and exon 1–3 boundaries appeared normal, indicating no alterations in this splice junction (Fig. 2D). These results indicate that the c. 142+5G>C variant may reduce/obliterate exon 1/intron 1 as a splice donor site. Without the presence of a highly predictive alternative splice donor site, it is possible that the mRNA of this *OPA3* allele is not or only sparsely spliced. It remains to be determined whether this variant, or another unidentified intronic or other regulatory variant underlies the decay of the allele with the wild type c.1 adenine nucleotide, which was not present in the cDNA sequence of Fig. 2C.

Our cellular analysis showed morphological alterations with increased fragmentation of the mitochondrial network in fibroblasts from Pt. 2. Changes in mitochondrial morphology and dynamics have previously been described in cells with altered *OPA3* [8], [10], [18]. Since we are not certain whether the c.1A>G variant allele will produce a protein product, we cannot speculate whether the altered mitochondrial morphology in our patient cells results from absent or truncated OPA3 protein expression. Altered mitochondrial morphology is also described in a series of other neurodegenerative disorders [22], [23], [24], suggesting a potential role of OPA3 in the regulation of mitochondrial morphology.

To date, the function of OPA3 remains unknown. The suspected mitochondrial pathology of OPA3 defects can explain the clinical–biochemical hallmarks of OPA3-related 3-MGA-uria; optic atrophy, neuropathy and increased urinary excretion of 3-MGA and 3-MGR. Optic atrophy and neuropathy result from apoptosis of cells that form or support nerves, most of which have high energy demands and have an elongated (axional) shape. Mitochondrial morphology, distribution and function are pivotal to survival of these cells, and optic atrophies and neuropathies are often associated with mitochondrial defects [11], [25]. Moderately increased 3-MGA and 3-MGR is a common finding in mitochondrial respiratory chain disorders [1], [2], [3], [4], [26], [27], and, in addition, most of the specified forms of 3-MGA-uria disorders affect mitochondrial proteins [4]. Note that the increase in 3-MGA and 3-MGR excretion is unique for OPA3-related optic atrophy [11].

## Figures and Tables

**Table 1:** Human *OPA3* variants.

Protein variant	cDNA variant	*OPA3* exon	Disorder	Ethnicity	Ref
p.M1V	c.1A>G	1	3-MGCA Type III	Indian	This report
p.L11Q	c.32 T>A	1	3-MGCA Type III	Pakistani	[15]
Unknown	c.142+5G>C	Intron 1	3-MGCA Type III	Indian	This report
Unknown	c.143-1G>C	Intron 1	3-MGCA Type III	Iraqi-Jewish	[7]
p.Q108_E113del	c.320_337del	2	3-MGCA Type III	Turkish-Kurdish	[13]
p.Q139X	c.415C>T	2	3-MGCA Type III	Indian	[14]
p.V3_G4insAP	c.10_11ins CGCCCG	1	ADOA	Unreported	[18]
p.G93S	c.277G>A	2	ADOA	French	[16]
p.Q105E	c.313C>G	2	ADOA	Unreported	[16]

## References

[1] Gunay-Aygun M. (2005). 3-Methylglutaconic aciduria: a common biochemical marker in various syndromes with diverse clinical features. Mol. Genet. Metab..

[2] Gibson K.M., Elpeleg O.N., Jakobs C., Costeff H., Kelley R.I. (1993). Multiple syndromes of 3-methylglutaconic aciduria. Pediatr. Neurol..

[3] Wortmann S.B., Duran M., Anikster Y., Barth P.G., Sperl W., Zschocke J., Morava E., Wevers R.A. (2013). Inborn errors of metabolism with 3-methylglutaconic aciduria as discriminative feature: proper classification and nomenclature. J. Inherit. Metab. Dis..

[4] Wortmann S.B., Kluijtmans L.A., Rodenburg R.J., Sass J.O., Nouws J., van Kaauwen E.P., Kleefstra T., Tranebjaerg L., de Vries M.C., Isohanni P., Walter K., Alkuraya F.S., Smuts I., Reinecke C.J., van der Westhuizen F.H., Thorburn D., Smeitink J.A., Morava E., Wevers R.A. (2013). 3-Methylglutaconic aciduria—lessons from 50 genes and 977 patients. J. Inherit. Metab. Dis..

[5] Costeff H., Elpeleg O., Apter N., Divry P., Gadoth N. (1993). 3-Methylglutaconic aciduria in “optic atrophy plus”. Ann. Neurol..

[6] Gunay-Aygun M., Huizing M., Anikster Y., Pagon R.A., Adam M.P., Bird T.D. (1993–2013). OPA3-related 3-methylglutaconic aciduria. GeneReviews™ (www.*genereviews*.org).

[7] Anikster Y., Kleta R., Shaag A., Gahl W.A., Elpeleg O. (2001). Type III 3-methylglutaconic aciduria (optic atrophy plus syndrome, or Costeff optic atrophy syndrome): identification of the OPA3 gene and its founder mutation in Iraqi Jews. Am. J. Hum. Genet..

[8] Huizing M., Dorward H., Ly L., Klootwijk E., Kleta R., Skovby F., Pei W., Feldman B., Gahl W.A., Anikster Y. (2010). OPA3, mutated in 3-methylglutaconic aciduria type III, encodes two transcripts targeted primarily to mitochondria. Mol. Genet. Metab..

[9] Schluter A., Fourcade S., Domenech-Estevez E., Gabaldon T., Huerta-Cepas J., Berthommier G., Ripp R., Wanders R.J., Poch O., Pujol A. (2007). PeroxisomeDB: a database for the peroxisomal proteome, functional genomics and disease. Nucleic Acids Res..

[10] Ryu S.W., Jeong H.J., Choi M., Karbowski M., Choi C. (2010). Optic atrophy 3 as a protein of the mitochondrial outer membrane induces mitochondrial fragmentation. Cell. Mol. Life Sci..

[11] Huizing M., Brooks B.P., Anikster Y. (2005). Optic atrophies in metabolic disorders. Mol. Genet. Metab..

[12] Ferre M., Bonneau D., Milea D., Chevrollier A., Verny C., Dollfus H., Ayuso C., Defoort S., Vignal C., Zanlonghi X., Charlin J.F., Kaplan J., Odent S., Hamel C.P., Procaccio V., Reynier P., Amati-Bonneau P. (2009). Molecular screening of 980 cases of suspected hereditary optic neuropathy with a report on 77 novel OPA1 mutations. Hum. Mutat..

[13] Kleta R., Skovby F., Christensen E., Rosenberg T., Gahl W.A., Anikster Y. (2002). 3-Methylglutaconic aciduria type III in a non-Iraqi-Jewish kindred: clinical and molecular findings. Mol. Genet. Metab..

[14] Ho G., Walter J.H., Christodoulou J. (2008). Costeff optic atrophy syndrome: new clinical case and novel molecular findings. J. Inherit. Metab. Dis..

[15] Arif B., Kumar K.R., Seibler P., Vulinovic F., Fatima A., Winkler S., Nurnberg G., Thiele H., Nurnberg P., Jamil A.Z., Bruggemann A., Abbas G., Klein C., Naz S., Lohmann K. (2013). A novel OPA3 mutation revealed by exome sequencing: an example of reverse phenotyping. JAMA Neurol..

[16] Reynier P., Amati-Bonneau P., Verny C., Olichon A., Simard G., Guichet A., Bonnemains C., Malecaze F., Malinge M.C., Pelletier J.B., Calvas P., Dollfus H., Belenguer P., Malthiery Y., Lenaers G., Bonneau D. (2004). OPA3 gene mutations responsible for autosomal dominant optic atrophy and cataract. J. Med. Genet..

[17] Verny C., Amati-Bonneau P., Dubas F., Malthiery Y., Reynier P., Bonneau D. (2005). An OPA3 gene mutation is responsible for the disease associating optic atrophy and cataract with extrapyramidal signs. Rev. Neurol. (Paris).

[18] Grau T., Burbulla L.F., Engl G., Delettre C., Delprat B., Oexle K., Leo-Kottler B., Roscioli T., Kruger R., Rapaport D., Wissinger B., Schimpf-Linzenbold S. (2013). A novel heterozygous OPA3 mutation located in the mitochondrial target sequence results in altered steady-state levels and fragmented mitochondrial network. J. Med. Genet..

[19] Davies V.J., Powell K.A., White K.E., Yip W., Hogan V., Hollins A.J., Davies J.R., Piechota M., Brownstein D.G., Moat S.J., Nichols P.P., Wride M.A., Boulton M.E., Votruba M. (2008). A missense mutation in the murine Opa3 gene models human Costeff syndrome. Brain.

[20] Ramalho A.S., Lewandowska M.A., Farinha C.M., Mendes F., Goncalves J., Barreto C., Harris A., Amaral M.D. (2009). Deletion of CFTR translation start site reveals functional isoforms of the protein in CF patients. Cell. Physiol. Biochem..

[21] Kozak M. (1989). The scanning model for translation: an update. J. Cell Biol..

[22] Olichon A., Baricault L., Gas N., Guillou E., Valette A., Belenguer P., Lenaers G. (2003). Loss of OPA1 perturbates the mitochondrial inner membrane structure and integrity, leading to cytochrome c release and apoptosis. J. Biol. Chem..

[23] Krebiehl G., Ruckerbauer S., Burbulla L.F., Kieper N., Maurer B., Waak J., Wolburg H., Gizatullina Z., Gellerich F.N., Woitalla D., Riess O., Kahle P.J., Proikas-Cezanne T., Kruger R. (2010). Reduced basal autophagy and impaired mitochondrial dynamics due to loss of Parkinson's disease-associated protein DJ-1. PLoS One.

[24] Misko A.L., Sasaki Y., Tuck E., Milbrandt J., Baloh R.H. (2012). Mitofusin2 mutations disrupt axonal mitochondrial positioning and promote axon degeneration. J. Neurosci..

[25] Itoh K., Nakamura K., Iijima M., Sesaki H. (2013). Mitochondrial dynamics in neurodegeneration. Trends Cell Biol..

[26] Ibel H., Endres W., Hadorn H.B., Deufel T., Paetzke I., Duran M., Kennaway N.G., Gibson K.M. (1993). Multiple respiratory chain abnormalities associated with hypertrophic cardiomyopathy and 3-methylglutaconic aciduria. Eur. J. Pediatr..

[27] Scaglia F., Sutton V.R., Bodamer O.A., Vogel H., Shapira S.K., Naviaux R.K., Vladutiu G.D. (2001). Mitochondrial DNA depletion associated with partial complex II and IV deficiencies and 3-methylglutaconic aciduria. J. Child Neurol..

